# Integrative Taxonomy Reveals a New Species of the Genus *Lejeunea* (Marchantiophya: Lejeuneaceae) from Peninsular Malaysia

**DOI:** 10.3390/plants11131642

**Published:** 2022-06-21

**Authors:** Gaik Ee Lee, Julia Bechteler, Tamás Pócs, Alfons Schäfer-Verwimp, Hung Yung Tang, Poh Wai Chia

**Affiliations:** 1 Faculty of Science and Marine Environment, Universiti Malaysia Terengganu, Kuala Nerus 21030, Malaysia; pohwai@umt.edu.my; 2 Institute of Tropical Biodiversity and Sustainable Development, Universiti Malaysia Terengganu, Kuala Nerus 21030, Malaysia; 3 Nees Institute for Biodiversity of Plants, University of Bonn, 53115 Bonn, Germany; bechteler@uni-bonn.de; 4 Botany Department, Institute of Biology, Eszterházy Károly Catholic University, Pf. 43, H-3301 Eger, Hungary; pocs.tamas33@gmail.com; 5 Mittlere Letten 11, 88634 Herdwangen-Schönach, Germany; moos.alfons@kabelbw.de; 6 Department of Geology, University of Malaya, Kuala Lumpur 50603, Malaysia; tanghungyung@gmail.com

**Keywords:** *Lejeunea malaysiana*, Lejeuneaceae, new species, Malaysia, integrative taxonomy

## Abstract

Prior to the advent of molecular work, the observable variation in vegetative reproduction has been used to classify *Lejeunea* into subgenera and sections. Thereby, the ability of developing caducous leaves was regarded as major factor. A reexamination of several *Lejeunea* specimens revealed that *L. cocoes* with caducous leaves shows considerable morphological differences with non-caducous leaved plants of *L. cocoes*. Phylogenetic analyses based on a three-marker dataset (*rbc*L, *trn*LF and the nuclear ribosomal ITS region) indicated two independent and robust lineages of the morpho-species *L. cocoes*. We consider both clades as two distinct species and therefore describe the new species, *L. malaysiana* for *L. cocoes* morpho-species with caducous leaves. *Lejeunea malaysiana* is characterized by its caducous leaves with ribbon-like and plantlet regenerants, strongly reduced leaf lobules, distant and deeply bilobed underleaves, long-keeled obovoid perianth, and autoicy and ranges from tropical Asia to the Pacific region.

## 1. Introduction

Malaysia has a very rich flora of liverworts with about 800 recorded species and infraspecific taxa [1]. The liverwort flora of Malaysia, particularly from the states of Sabah and Sarawak, has been much explored since the 19th century; however, many mountains and forests in Malaysia remain largely unexplored and many taxa are completely unknown [2]. Papers dealing with Malaysian liverworts are being published each year, including new floristic liverwort explorations, e.g., [3,4,5], descriptions of new species, e.g., [6,7,8], and integrative morphological and molecular studies, e.g., [9,10,11]. Here, we describe another new species of *Lejeunea* from the state of Pahang in Peninsular Malaysia based on morphological and molecular evidence.

A comprehensive taxonomic revision of the genus *Lejeunea* in Malaysia was published by Lee [12], leading to the recognition of 30 species and two varieties in this country. New records of *Lejeunea* continue to be discovered, and currently, 37 species and two varieties are reported from Malaysia, of which three are endemic, viz., *L. contracta* Mizut., *L. gradsteinii* G.E. Lee et al. and *L. pectinella* Mizut. [13]. The genus *Lejeunea* is one of the largest genera of the family Lejeuneaceae with about 200 to 300 species [14,15]. The molecular dating results have unveiled the origin and biogeographic history of *Lejeunea* in which the genus attained its greatest diversity during the Miocene period [16]. The genus is well known for its extensive morphological homoplasy and therefore morphology alone is inadequate to classify the genus. Only an integrative taxonomical approach combining molecular and morphological data can help to untangle species complexes and establish more natural species circumscriptions, e.g., [17]. 

In this study, we incorporated molecular data based on sequences of two chloroplast regions (*rbc*L, *trn*LF) and the nuclear ribosomal ITS region of multiple accessions morphological circumscribed species of *L. cocoes* Mitt. Our phylogenetic analyses (RAxML, MrBayes), in combination with extensive morphological reevaluations of the specimens, discovered two independent monophyletic lineages of the morpho-species *L. cocoes*: three accessions of *L. cocoes* with caducous leaves grouped together, whereas two accessions of *L. cocoes* without caducous leaves formed another independent lineage. We consider both clades as two biologically different entities and not closely related and therefore describe a new species for *L. cocoes* morpho-species with caducous leaves. 

## 2. Results

### 2.1. Morphology

Morphological study of 10 specimens initially assigned to *L. cocoes* from Peninsular Malaysia, Indonesia, Chagos Archipelago (Indian Ocean) and the Fiji Islands revealed that they have important differences in comparison to the typical *L. cocoes*, a tropical Asian species, including the vegetative reproduction by means of caducous leaves with ribbon-like and plantlet regenerants, strongly reduced leaf lobules, long-keeled obovoid perianth (up to 3/4 of the perianth length), and autoicy. However, on the other hand, both species are morphologically very similar and vary profusely in the absence or presence of caducous leaves, which are so far only regarded as a means of vegetative reproduction. 

### 2.2. Molecular Phylogeny

**Figure 1:**
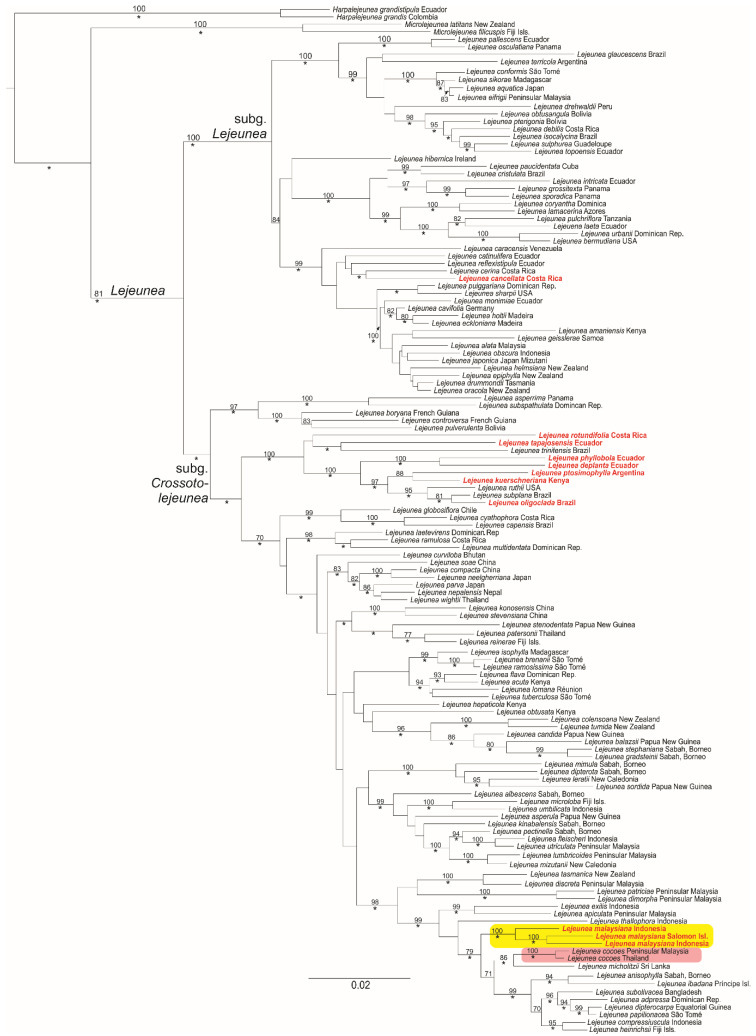
Bayesian Inference (BI) phylogeny of *Lejeunea* based on three markers from nuclear and plastid DNA. Bayesian Posterior Probability (BPP) ≥ 0.95 is indicated by a star; maximum likelihood probabilities ≥ 70 are shown at branches. Accessions of *L. malaysiana* (initially assigned to *L. cocoes*), highlighted in yellow, are grouped together with high support (BS 100, BPP 1) whereas accessions of *L. cocoes* (without caducous leaves), highlighted in red, formed another independent lineage. Species with caducous leaves are in red font.

The Bayesian and Maximum Likelihood tree topology are identical and split into two well-supported main clades (Figure 1). Species with caducous leaves and other means of vegetative reproduction, e.g., regenerants, cladia, and fragmenting stem and branches, are diffusely distributed and are found in both main lineages of *Lejeunea.* The three accessions were initially assigned to *L. cocoes* that have caducous leaves grouped with high support (BS 100, BPP 1), whereas two accessions of *L. cocoes* without caducous leaves formed another independent lineage (BS 100, BPP 1) in a well-supported clade including *L. micholitzii* Mizut. (BS 86, BPP 1). The latter two species are found in a sister relationship to a clade comprising autoicous species including *L. anisophylla* Mont., *L. ibadana* A.J. Harr. and E.W. Jones, *L. subolivacea* Mizut., *L. adpressa* Nees, *L. dipterocarpa* E.W. Jones, *L. papilionacea* Prantl, *L. compressiuscula* (Steph.) G.E. Lee and Heinrichs, and *L. heinrichsii* G.E. Lee et al. Based on our molecular phylogeny and morphological investigation, we describe the *L. cocoes* morpho-species with caducous leaves as a new species, *L. malaysiana*. The specific epithet ‘malaysiana’ refers to the type locality in Malaysia. 

### 2.3. Taxonomic Description

**Figure 2:**
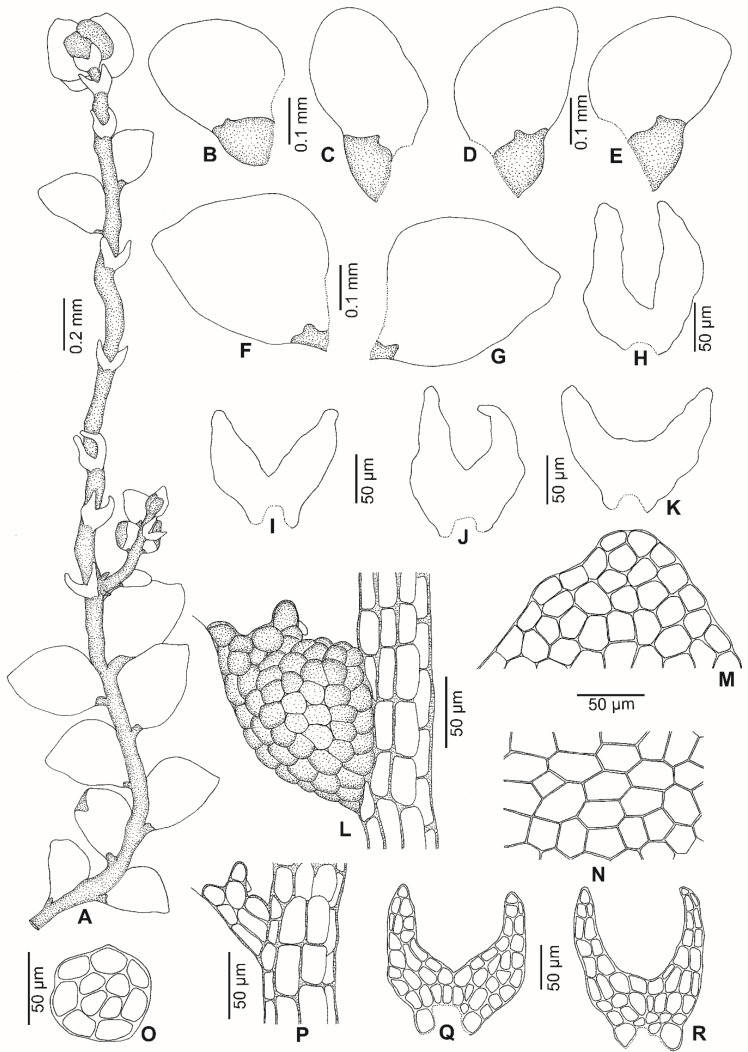
*Lejeunea malaysiana* G.E. Lee and Pócs, *sp. nov.* (**A**) Part of plant. (**B**–**E**) Leaves. (**F**,**G**) Caducous leaves. (**H**–**K**) Underleaves. (**L**) Leaf lobule. (**M**) Apical cells of leaf lobe. (**N**) Basal cells of leaf lobe. (**O**) Cross-section of the stem. (**P**) Leaf lobule (reduced). (**Q**,**R**) Cells of underleaves. All were drawn from the holotype, *G.E. Lee and H.Y. Tang 11001* (UMTP).

**Figure 3:**
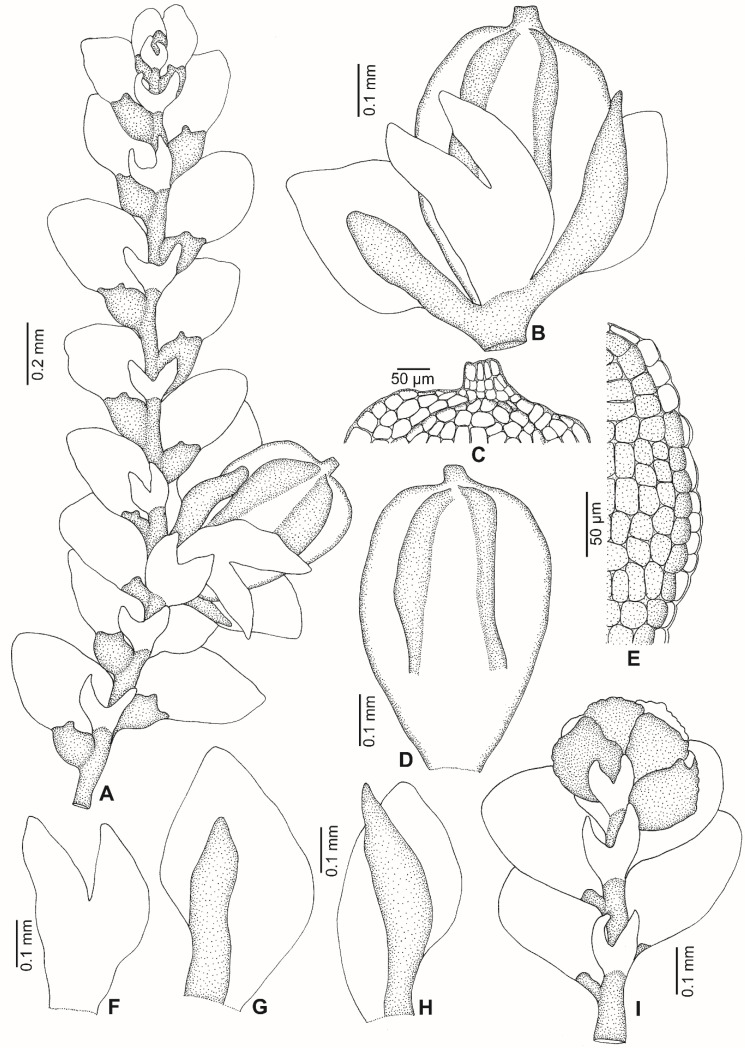
*Lejeunea malaysiana* G.E. Lee and Pócs, *sp. nov*. (**A**) Part of plant, with the perianth-bearing branch. (**B**) Perianth with bracts and bracteole. (**C**) Apical cells of the perianth. (**D**) Perianth. (**E**) Cells at the lateral keel of the perianth. (**F**) Female bracteole. (**G**,**H**) Female bracts. (**I**) Androecial shoot. All were drawn from the holotype, *G.E. Lee and H.Y. Tang 11001* (UMTP).

**Figure 4:**
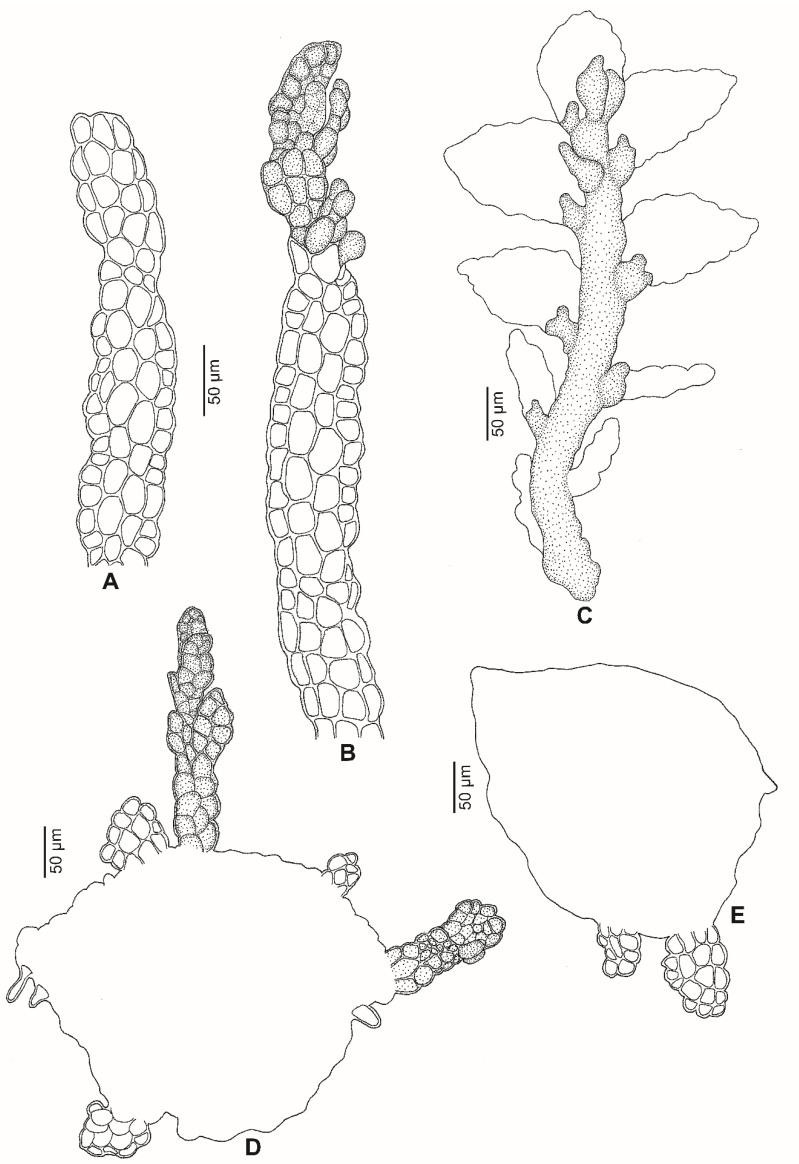
Vegetative reproduction of *Lejeunea malaysiana* G.E. Lee and Pócs, *sp. nov*. (**A**) Ribbon-like regenerant. (**B**) Young plantlet on top of the ribbon-like regenerant. (**C**) Mature plantlet, fall off from the caducous leaf. (**D**,**E)** Ribbon-like and young plantlets on marginal cells of caducous leaves. All were drawn from the holotype, *G.E. Lee and H.Y. Tang 11001* (UMTP).

*Lejeunea malaysiana* G.E. Lee and Pócs, *sp. nov.* (Figure 2, Figure 3 and Figure 4).

Type: Peninsular Malaysia, Pahang, Genting Highlands, along the forest trail to the Goh Tong Jaya station near the waterfall, at 845 m elevation, 6 May 2011, *G.E. Lee and H.Y. Tang 11001* (holotype: UMTP 01730; isotype: UKMB).

Diagnosis: Affinity to the tropical Asian *L. cocoes* Mitt. in the ovate leaves with narrowly rounded apices, smooth cuticles, laminal cells with indistinct trigones and without intermediate thickenings, and distantly arranged underleaves with divergent lobes but differs by its caducous leaves, strongly reduced leaf lobules, long-keeled obovoid perianths and autoicy. 

Plant autoicous, 0.6–0.7 mm wide, irregularly branched. *Stem* ca. 50 µm diameter, about 4–5 cells high in cross-section, epidermal cells 7, 20–38 µm wide, medullary cells 4–5, 15–25 µm wide. *Leaf lobes* distant to contiguous, 0.30–0.45 × 0.20–0.35 mm, ovate to ovate-oblong; leaf apex narrowly rounded, always flat; leaf margin weakly crenulate due to projecting cells; the ventral margin forming an angle of 150°–170° with the keel when flattened; insertion line about 8–10 lobe cells. *Leaf cells* quadrate to hexagon; apical cells 18–25 × 13–20 µm, median cells 25–30 × 20–25 µm, basal cells 25–38 × 20–25 µm; cell walls hyaline with small or indistinct trigones and without intermediate thickenings; cuticle smooth; oil bodies not seen. *Leaf lobules* strongly reduced, ca. 0.1 × 0.07–0.1 mm, ovate, keel curved, free margin incurved partially; the hyaline papilla on the proximal side of the first tooth; the cell below the first tooth is of the same size as the first tooth, undifferentiated, 20–25 × 14–20 µm. *Underleaves* 0.12–0.15 × 0.10–0.15 mm, to 2–3 times wider than the stem, distant, ovate to orbicular (as long as wide), covering 1/4 of the leaf lobules; bilobed, lobes to 2/3–3/4 of underleaf length, about four cells wide; sinus broad, acute to obtuse, U to widely V-shaped; underleaf margin slightly crenulate; the two large basal underleaf cells differentiated; base straight. *Androecia* terminal on short branch or main shoots; male bracts in 3–4 pairs; male bracteole 1–2, same size as the underleaf; margin slightly crenulate; antheridia not seen. *Gynoecia* on short lateral branches or terminal on main shoots, with 1 (–2) innovations, with 1–2 gynoecia in a lateral position. *Female bracts* smaller than the leaf, erect-spreading when moist, not enveloping the perianth; lobes 0.45–0.50 × 0.25–0.30 mm, elliptical, apex narrowly rounded, margin slightly crenulate; lobules 0.25–0.30 × 0.08–0.10 mm, strongly reduced, oblong, apex acute, keels straight, smooth, ca. 0.15 mm long. *Female bracteoles* 0.3–0.4 mm × 0.15–0.20 mm, oblong with tips obtuse; lobes to 1/2 of female bracteole length, approximate to distant; sinus narrow, acute, unequally bifid; margin slightly crenulate. *Perianths* 0.60–0.65 × 0.3–0.4 mm; emergent up to 1/3 of the perianth length; obovoid, with 5 keels; beak 2–3 cells long; cells of the perianth at the keels mammillose and often winged, regularly rounded to quadrate, occasionally with relatively small trigones and without intermediate thickenings; stalk-like elongation lacking. *Sporophyte* not seen. *Vegetative propagules* by means of caducous leaves with ribbon-like and plantlet regenerants; caducous leaves ovate-triangular, 0.3–0.5 × 0.2–0.3 mm, lobules strongly reduced. 

Distribution and habitat: Known from Peninsular Malaysia, Indonesia, Chagos Archipelago (Indian Ocean), and the Fiji Islands, growing on tree trunks from lowland to montane rainforest, 100–1300 m elevation. 

Further specimens examined: MALAYSIA. Pahang: Genting Highlands, along the forest trail to Goh Tong Jaya station near the waterfall, 845 m elevation, 6 May 2011, *G.E. Lee and H.Y. Tang 11002, 11003* (UMTP 01731, 01732). INDONESIA. Bali: Westbali, Distr. Jembrana, Bergland zwischen Pekutatan und Pucaksari (westlich Pupuan), Kulturland bei Pucaksari, epiphytisch an *Eugenia aromatica*, 730 m, 2 June 1995, *Schäfer-Verwimp and Verwimp 16892* (JE, GOET, FR); Ostbali, Distr. Amlapura, Südseite des Gunung Agung, im Dorf Selat epiphytisch an Kokospalme, 440 m, 1 June 1999, *Schäfer-Verwimp and Verwimp 21050* (JE, GOET, FR, CAS). INDIAN OCEAN. Chagos Archipelago: *Seaward 108088* (JE), Salamon Atoll and Diego Garcia Island, *Seaward 108028b*, *112677112679* (MRDS, EGR). FIJI. Southern coast of Viti Levu Island (Coral Coast) in the high rainfall area, Tambua Sands Beach Resort E of Namada village, in coastal coconut stand, at palm base, 3–5 m, 18°11.472′ S, 177°37.582′ E, *S. and T. Pócs 03255/W* (EGR); S tip of Taveuni Island, in the crater of the extinct Tavuyagea volcano, relatively dry *Barringtonia edulis* dominated lowland rainforest on the outer slopes, 150–295 m, 16°59.660–662′ S, 179°55.296–360′ E *S. and T. Pócs 03293/U* (EGR); Central part of Kadavu (Kandavu) Island, 2–3 km NNE of Vunisea, along the Namara Road, secondary lowland rainforest on lilac volcanic soil, on the slopes at 120–165 m, 19°01.731–880′ S, 178°10.265–750′ E, *S. and T. Pócs 03300/G* (EGR).

## 3. Discussion

Integrative taxonomy includes information from different types of data and methodologies; in the present study, molecular data aimed to establish a more robust species hypothesis and circumscription. Particularly for the genus *Lejeunea*, integration of molecular and morphological data should therefore be encouraged, because only such combined molecular–morphological studies allow for a reexamination of morphological characters that were previously considered to be of diagnostic importance in the species circumscriptions. Recent studies have demonstrated congruence or conflict between molecular and morphological data in *Lejeunea*, e.g., molecular phylogenetic analyses resolved three morphologically similar species, i.e., the African *Taxilejeunea pulchriflora* Pearson and two Asian *L. propagulifera* Gradst. and *L. tamaspocsii* G.E.Lee, in a robust monophyletic lineage [9]. In comparison, morphologically similar accessions initially assigned to *L. tumida* Mitt. belong to four species nested in different clades [18]. Before molecular data, diagnostic characters such as toothed or eplicate perianths were used for generic distinction or subgenera delimitations. Such morphological data were often proved to be homoplastic in phylogenetic analyses [9,19]. In the present study, one such morphological character is caducous leaves which were regarded as one characteristic for the species *L. cocoes*. In our phylogenetic analyses, however, we discovered two independent monophyletic lineages that by reexamination of the plant material turned out to be linked to the presence or absence of caducous leaves on specimens of the morpho-species of *L. cocoes*. We thus consider both clades as two different species and introduce the new species *L. malaysiana* for the clade including plants with caducous leaves. 

Generally, vegetative reproduction of *Lejeunea* is classified into five types, viz., plantlet regenerants, ribbon-like propagules, cladia, caducous, and fragmenting stem or branches [12,20]. Regenerants and propagules that grow on leaf margins are very common forms of vegetative reproduction and have previously been described in *Lejeunea* [9,20,21,22,23,24]. The cladia type thus far produces exclusively on *L. dimorpha* T.Kodama, an obligate epiphyllous taxon reported from Vietnam, Peninsular Malaysia, Borneo, Papua New Guinea and the Philippines [25]. The caducous type may occur on the leaves, leaf lobes, underleaves, or branches, but caducous leaves is the most common and has been reported in many species of *Lejeunea*, e.g., in the Neotropics: *L. deplanata* Nees, *L. phyllobola* Nees and Mont., *L. oligoclada* Spruce, *L. perpapillosa* M.E.Reiner and K.C.Pôrto, *L. parviloba* Ångstr., *L. flagellifera* Bastos et al.; in tropical America: *L. cochleata* Spruce, *L. ptosimophylla* C. Massal., *L. rionegrensis* Spruce [20,26]; in Africa: *L. kuerschneriana* Pócs [27], and in tropical Asia: *L. subacuta* Mitt. [28,29]. The caducous leaves of the species mentioned above are occasionally with plantlet regenerants on the marginal leaf cells, e.g., [30] (p. 14), whereas in *L. malaysiana*, apart from the leaf margin, the plantlet regenerants are also seen growing on the top of long ribbon-like regenerants. Two different regenerants are developed on the caducous leaves of *L. malaysiana*: the young plantlets grow on top of the long ribbon-like regenerants (10–24(30) cells or 0.2–0.6 mm long and 4–5 cells wide) whereas other ribbon-like regenerants (4–6 cells long and 4–5 cells wide) developing on the marginal leaf cells and grow into young plantlets. Each caducous leaf of *L. malaysiana* may produce 2(–6) regenerants and the leaf lobes produced on the stem are hardly found. However, the gynoecial shoot is usually is present with all its leaves. Although vegetative reproduction plays a vital role in the range size of liverworts and increases the chances of successful long-range dispersal in species with unisexual populations, only a few species of *Lejeunea* reproduce frequently by vegetative reproduction. In the molecular phylogeny of *Lejeunea*, we included 24 species, including *L. malaysiana*, with vegetative reproduction and found them diffusely distributed throughout the two subgenera of *Lejeunea* (Figure 1). Out of 24 species, only seven (*L. deplanata* Nees, *L. malaysiana*, *L. kuerschneriana* Pócs, *L. oligoclada* Spruce, *L. phyllobola* Nees and Mont., *L. ptosimophylla* C. Massal, and *L. tapajosensis* Spruce) are with the occurrence of caducous leaves and are nested in the subg. *Crossotolejeunea* but they do not form a monophyletic group. All but *L. malaysiana* are nested in a robust monophyletic lineage with the Neotropics accessions of *L. rotundifolia* Mitt., *L. trinitensis* Lindenb., *L. ruthii* (A. Evans) R.M. Schust., and *L. subplana* (Steph.) C.J. Bastos. However, vegetative reproduction by caducous leaves is not known in the latter four species. In our molecular analyses, we also included *L. cancellata* (Steph.) C.J. Bastos, which can produce caducous branches, but this species is nested in the largely neotropical clade, subg. *Lejeunea*. 

*Lejeunea malaysiana* and *L. cocoes* share similar characteristics, including the cells with relatively small or poorly developed trigones and without intermediate thickenings, the smooth cuticle, the small and bilobed underleaves with lanceolate lobes, and the obovoid perianths. Indeed, *L. malaysiana* closely approaches *L. cocoes* in these features and plants of *L. malaysiana* not showing vegetative reproduction can be mistaken for *L. cocoes*. The development of caducous leaves is often abundant in *L. malaysiana* and is usually evident even on casual examination, unlike in other *Lejeunea* species (except for *L. dimorpha* [25]) where the propagules are more sporadic and infrequent. Vegetative reproduction of *L. cocoes* is sparsely produced and exclusively by means of ribbon-like regeneration, never by caducous leaves. Equally distinctive is the autoicy, which separates *L. malaysiana* from *L. cocoes*. In our molecular phylogenetic analyses, both species are resolved in different monophyletic lineages where *L. cocoes* forms a sister relationship with *L. micholitzii*. *Lejeunea malaysiana* is perhaps most closely related to *L. subacuta* reported from India and China, sharing with it a strong ability to produce caducous leaves; however, *L. subacuta* differs from *L. malaysiana* in the dioicy, oblong leaf lobe with occasionally marginal rhizoids, and presence of male bracteoles throughout the androecial shoot [28,29].

## 4. Materials and Methods

### 4.1. Plant Materials and Morphological Investigation

Morphological characteristics of *L. malaysiana* were studied based on herbarium specimens from the Eszterházy Károly College herbarium (EGR), the Conservatoire et Jardin Botanique de la Ville de Genève (G), the Göttingen University herbarium (GOET), the herbarium of Universiti Kebangsaan Malaysia (UKMB), and the herbarium of Universiti Malaysia Terengganu (UMTP). The holotype is deposited at the herbarium UMTP under the management of South China Sea Repository and Research Centre, Institute of Oceanography and Environment, Universiti Malaysia Terengganu. Plant material was examined by light microscopy and the drawing of the type specimen was produced using an Olympus BX43 microscope equipped with a drawing tube. 

### 4.2. DNA Extraction, PCR Amplification, Sequencing and Alignment

Gametophytic plant tissue was isolated from five dried herbarium specimens initially identified as *L. cocoes*. Total genomic DNA was isolated using the Invisorb Spin Plant Mini Kit (Stratec Molecular GmbH, Berlin, Germany) prior to amplification. Amplification of the three molecular genetic markers, *rbc*L, *trn*LF, and ITS was carried out with the PCR protocol [9,31]. The newly generated sequences were assembled and edited using PhyDE v0.9971 and then compared to Genbank sequences using nucleotide BLAST search [32] to evaluate their assumed belonging to the genus *Lejeunea*. The new sequences were then aligned manually and integrated into the *Lejeunea* alignment of Lee et al. [16] using BioEdit 5.0.9 [33]. Taxa used in the study, including GenBank accession numbers and voucher details, are listed in Table S1. Ambiguous hotspot positions were excluded and the reverse complement of one inverted repeat in the *trn*LF region was aligned with the other sequences prior to molecular phylogenetic analyses [34]. 

### 4.3. Phylogenetic Analyses

Maximum Likelihood (ML) analysis was conducted with RAxML-HPC 8.2.8 [35]. Appropriate DNA partitions and corresponding substitution models, rate of invariable sites, and gamma rate heterogeneity were identified using JModelTest 2 [36] and the Akaike information criterion (AIC). This resulted in TVM + I + G for *rbc*L, TVM + G for *trn*L-*trn*F, and TIM1 + I + G for nrITS. Since these models were not available in RAxML, the best fitting overparameterized model GTR + G was used. Trees were generated by selecting 10 independent runs and the multi-parametric bootstrap option autoMRE, resulting in 300 bootstrap replicates to compute node support as bootstrap values at each node. The compatibility of the chloroplast and nuclear regions was explored by comparing the trees obtained from independent ML analyses for each region. The trees were visually compared to identify conflicting nodes with BV higher than 70% [37], and an ML analysis was conducted with two chloroplasts (*rbc*L, *trn*LF) partitions and a nuclear (ITS) partition. 

Bayesian Inference (BI) was undertaken in MrBayes 3.2.6 [38]. The same dataset, nuclear substitution models, and partitions as those in the ML analysis were used. Two metropolis-coupled Markov chain Monte Carlo (MCMC) analyses, including three heated chains and one cold chain, were run for two million generations and sampled every 200 generations. Convergence and stationarity of the runs were checked in TRACER 1.7 and an average standard deviation (SD) of split frequency below 0.01 indicated a sufficiently long run. The first 25% of the trees were discarded as burn-in and the remaining trees were summarized by TreeAnnotator 1.8.3 [39] using median node heights. The resulting maximum clade credibility (MCC) tree was visualized in FigTree 1.4.3. Nodes with posterior probability values ≥ 0.95 were regarded as good support [40]. 

## 5. Conclusions

Our present study shows a strong conflict between molecular phylogenies and morphology-based classification in *Lejeunea* and its lineages. The apparent incongruence between the molecular variation and the morphologically circumscribed taxa is primarily demonstrated by extensive homoplasy in *Lejeunea* where most previously assumed morphological characters appear to be highly homoplastic. Hence, the natural classification of *Lejeunea* will require integrative taxonomical studies, including comprehensive taxonomic revision and DNA sequence data. These data are essential for evaluating different species concepts and exploring the morphological and genetic variation in reconstructing relationships and the circumscriptions of *Lejeunea* species.

## Data Availability

No additional data are available.

## Supplementary Materials

The following supporting information can be downloaded at: https://www.mdpi.com/article/10.3390/plants11131642/s1, Table S1: Taxa used in the present study with information about the origin of the studied material, vouchers, as well as GenBank accession numbers.
