# Supplementary material for: Integrative Taxonomy Reveals a New Species of the Genus Lejeunea (Marchantiophya: Lejeuneaceae) from Peninsular Malaysia
Source: Plants (Basel). 2022 Jun 21;11(13):1642. doi: 10.3390/plants11131642 (PMC9269079; doi:10.3390/plants11131642)
Supplement: Supplementary file 1 [file plants-11-01642-s001.zip › plants-1744000-supplementary.pdf]

Table S1. Taxa used in the present study with information about the origin of the studied material, vouchers, as well as GenBank accession numbers are included. New sequences in boldface.

Taxon	Origin	Collector	GenBank Accession No.		
			*rbc*L	*trn*LF	ITS
Harpalejeunea grandis Grolle & M.E.Reiner	Colombia	Cleef 6450 (GOET)	KC313144	KC313184	KC313106
H. grandistipula R.M.Schust.	Ecuador	Schäfer-Verwimp et al. 24163/B (GOET)	KC313145	KC313185	KC313107
Lejeunea acuta Mitt.	Kenya	Chuah-Petiot Mb 22 (JE)	KF556384	KF556134	KF555917
L. adpressa Nees	Dominican Rep.	Schäfer-Verwimp & Verwimp 26931/B (GOET)	KF556386	KF556136	KF555919
L. alata Gottsche	Malaysia	Schäfer-Verwimp & Verwimp 18912 (GOET)	-----------	KF556140	KF555922
L. geisslerae Pócs	Samoa	Bartlett 32262b (JE)	MN822951	MN823014	MN818974
L. albescens (Steph.) Mizut.	Sabah, Borneo	G.E.Lee 1533 (UKMB)	MN822952	MN823015	MN818975
L. amaniensis E.W.Jones	Kenya	Malombe & Chituyi 5006/Si.1aS5 (EGR)	KF556392	KF556143	KF556603
L. anisophylla Nees & Mont.	Sabah, Borneo	G.E.Lee 2145 (UKMB)	MN822953	MN823016	MN818976
L. apiculata Sande Lac.	Peninsular Malaysia	Pócs & Pócs 1003/AC (EGR)	MN823003	MN823017	MN818977
L. aquatica Horik.	Japan	Higuchi 1021 (JE)	-----------	KF556155	KF555933
L. asperrima Spruce	Panama	Schäfer-Verwimp & Verwimp 30817 (GOET)	KF556402	KF556157	KF555935
L. asperula (Steph.) Mizut.	Papua New Guinea	Streimann 40815 (JE)	-----------	KF556156	KF555934
L. bermudiana (A.Evans) R.M.Schust.	USA	Shaw 14939 (DUKE)	-----------	KF556158	KF555936
L. boryana Mont.	French Guiana	Holz FG 00-0103 (GOET)	KF556405	KF556159	KF555938
L. brenanii E.W.Jones	São Tomé	Shevock 39785 (M)	MN822954	MN823018	MN818978
L. cancellata Nees & Mont.	Costa Rica	Schäfer-Verwimp & Holz SV/H- 0507/C (GOET)	KF556409	KF556164	KF555942
L. capensis Gottsche	Brazil	Schäfer-Verwimp & Verwimp 15057 (GOET)	-----------	KF556167	KF555943
L. caracensis Lindenb.	Venezuela	Picón et al. 00227/CD (EGR)	MN822955	MN823019	MN818979
L. catinulifera Spruce	Ecuador	Wilson et al. 04-01 (GOET)	DQ983687	DQ987432	DQ987328
L. cavifolia (Ehrh.) Lindb.	Germany	Heinrichs 3695 (GOET)	AY548102	DQ238581	DQ987259
L. cerina (Lehm. & Lindenb.) Gottsche et al.	Costa Rica	Schäfer-Verwimp & Holz SV/H- 0471 (GOET)	KF556425	KF556180	KF555955
L. cocoes Mitt.	Peninsular Malaysia	G.E.Lee 2339 (UKMB)	MN822957	MN823021	MN818963 (ITS1)
***L. cocoes* Mitt.**	**Thailand**	**G.E.Lee 2496 (UKMB)**	**ON646222**	**ON646224**	-----------
L. colensoana (Steph.) M.A.M.Renner	New Zealand	Renner 300140 (AK)	-----------	JF308578	JF308549
L. compacta (Steph.) Steph.	China	Long 18826 (E)	MN822956	MN823020	MN818980
L. compressiuscula (Steph.) G.E.Lee & Heinrich	Indonesia	Schäfer-Verwimp & Verwimp 24923/E (GOET)	KF556519	KF556290	KF556050
L. conformis Nees & Mont.	São Tomé	Shevock 34483 (EGR)	MN822958	MN823022	MN818981
L. controversa Gottsche	French Guiana	Hartmann et al. 04-033 (GOET)	KF556432	KF556189	KF555964
L. corynantha Spruce	Dominica	Schäfer-Verwimp & Verwimp 17961/A (GOET)	MN995825	MN995826	MN990423

L. cristulata (Steph.) M.E.Reiner & Goda	Brazil	Giancotti 17 (JE)	-----------	KF556193	KF555966
L. curviloba Steph.	Bhutan	Long 10611 (JE)	-----------	KF556195	KF555967
L. cyathophora Mitt.	Costa Rica	Schäfer-Verwimp & Holz SV/H- 229 (M)	MN822959	MN823023	MN818982
L. debilis (Lehm. & Lindenb.) Nees & Mont.	Costa Rica, La Gamba	Schluder 7 (GOET)	KF556437	KF556197	KF555969
L. deplanata Nees	Ecuador, Pichincha	Schäfer-Verwimp et al. 24502/C (GOET)	KF556439	KF556199	KF555971
L. dimorpha Kodama	Peninsular Malaysia	G.E.Lee 2211 (UKMB)	MN822960	MN823024	MN818983
L. dipterocarpa E.W.Jones	Equatorial Guinea	Müller B316 (EGR)	MN822961	MN823025	MN818984
L. dipterota (Eifrig) G.E.Lee	Sabah, Borneo	G.E.Lee 1802 (UKMB)	MN822962	MN823026	MN818985
L. discreta Lindenb.	Peninsular Malaysia	G.E.Lee 2328 (UKMB)	MN822963	MN823027	MN818986
L. drehwaldii Heinrichs & Schäf.-Verw.	Peru	Drehwald 4384 (JE)	KF556445	KF556207	KF555978
L. eckloniana Lindenb.	Madeira	Stech 04-433 (L)	KF556447	KF556211	KF555983
L. eifrigii Mizut.	Peninsular Malaysia	G.E.Lee 2334 (UKMB)	MN822964	MN823028	MN818987
L. exilis (Reinw. et al.) Grolle	Indonesia	Schäfer-Verwimp & Verwimp 25231 (GOET)	KF556449	KF556213	KF555985
L. flava (Sw.) Nees	Dominican Rep.	Schäfer-Verwimp & Verwimp 26855/B (GOET)	KF556479	KF556243	KF556009
L. fleischeri (Steph.) Mizut.	Indonesia	Schäfer-Verwimp & Verwimp 24809/A (GOET)	MN822965	MN823029	MN818988
L. glaucescens Gottsche	Brazil	Schäfer-Verwimp & Verwimp 9724 (M)	MN822966	MN823060	MN818989
L. globosiflora (Steph.) Steph.	Chile	Gradstein 12418 (GOET)	MN822967	MN823030	MN818962/ MN818960
L. gradsteinii G.E.Lee et al.	Sabah, Borneo	G.E.Lee 1885 (UKMB)	MN822968	MN823031	MN818990
L. grossitexta (Steph.) M.E.Reiner & Goda	Panama	Schäfer-Verwimp & Verwimp 31000 (GOET)	KF556491	KF556256	KF556020
L. helmsiana Steph.	New Zealand	Renner 300069 (AK)	-----------	JF308569	JF308540
L. hepaticola Steph.	Kenya	Pócs & Pócs 04004/K (EGR)	MN822969	MN823032	MN818991
L. hibernica Grolle	Ireland	Long 11743 (JE)	-----------	KF556257	KF556021
L. holtii Spruce	Madeira	Drehwald & Drehwald 3719 (GOET)	KF556492	KF556258	KF556022
L. ibadana A.J.Harr & E.W.Jones	Príncipe Isl.	Shevock 42309 (EGR)	MN822970	MN823033	MN818992
L. intricata J.B.Jack & Steph.	Ecuador	Schäfer-Verwimp & Nebel 33217 (GOET)	-----------	-----------	KF556023
L. isocalycina (Nees) Spruce	Brazil	Costa & Gradstein 3720 (GOET)	KF556496	KF556262	KF556027
L. isophylla E.W.Jones	Madagascar	Lübenau 21 (EGR)	KF556497	KF556263	KF556028
L. japonica Mitt.	Japan	Mizutani 15618 (L)	KF556499	KF556265	KF556030
L. kinabalensis Mizut.	Sabah, Borneo	G.E.Lee 2138 (UKMB)	MN822971	MN823034	MN818993
L. konosensis Mizut.	China	Long & Shevock 37403 (E)	MN822972	MN823035	MN818994

L. kuerschneriana Pócs	Kenya	Chuah et al. 3017 (EGR)	MN822973	MN823036	MN818995
L. laeta (Lehm. & Lindenb.) Gottsche et al.	Ecuador	Schäfer-Verwimp et al. 24412 (GOET)	-----------	KF556267	KF556032
L. laetevirens Nees & Mont.	Dominican Rep.	Schäfer-Verwimp & Verwimp 27079 (GOET)	KF556508	KF556275	KF556037
L. lamacerina (Steph.) Schiffn.	Azores	Schäfer-Verwimp & Verwimp 29394 (GOET)	KF556510	KF556279	KF556041
L. leratii (Steph.) Mizut.	New Caledonia	Larrín 35964 (EGR)	MN822974	MN823062	MN818996
L. lomana E.W.Jones	Réunion	Pócs 08064/L (EGR)	KF556388	KF556138	KF555921
L. lumbricoides (Nees) Nees	Peninsular Malaysia	G.E.Lee 1429 (UKMB)	MN822975	MN823037	MN818997
L. micholitzii Mizut.	Sri Lanka	Schäfer-Verwimp & Verwimp 16374 (M)	MN822976	MN823063	MN818998
L. malaysiana G.E. Lee & Pócs	Indian Ocean	Seaward 108088 (JE)	-----------	KF556187	KF555962
L. malaysiana G.E. Lee & Pócs	Indonesia	Schäfer-Verwimp & Verwimp 21050 (GOET)	KF556430	KF556186	KF555961
***L. malaysiana* G.E. Lee & Pócs**	**Indonesia**	**Schäfer-Verwimp & Verwimp 16892 (GOET)**	**ON646223**	**ON646225**	**ON705052**
L. microloba Taylor	Fiji Isls.	Pócs 08013/Y (EGR)	KF556520	KF556291	KF556051
L. mimula Hürl.	Sabah, Borneo	G.E.Lee 1749 (UKMB)	MN822977	MN823038	MN818999
L. mizutanii Grolle	New Caledonia	von Konrat s.n. (EGR)	MN822978	MN823039	MN819000
L. monimiae (Steph.) Steph.	Ecuador	Schäfer-Verwimp & Preussing 23226/A (GOET)	KF556526	KF556298	KF556055
L. multidentata M.E.Reiner & Mustelier	Dominican Rep.	Pócs & Pócs 03157/A (EGR)	KF556527	KF556299	KF556056
L. neelgherriana Gottsche	Japan	Higuchi 0791954 (L)	-----------	KF556301	KF556058
L. nepalensis (Steph.) H.A.Mill. et al.	Nepal	Long 17250 (JE)	-----------	KF556302	KF556059
L. obscura Mitt.	Indonesia	Schäfer-Verwimp & Verwimp 24957/C (M)	MN822980	MN823041	MN819002
L. obtusangula Spruce	Bolivia	Krömer 869 (GOET)	KF556532	KF556307	KF556063
L. obtusata Gottsche	Kenya	Pócs et al. 04042/G (EGR)	MN822979	MN823040	MN819001
L. oligoclada Spruce	Brazil	Schäfer-Verwimp & Verwimp 13590 (GOET)	KF556533	KF556308	KF556064
L. oracola M.A.M.Renner	New Zealand	Renner 300078 (AK)	-----------	JF308571	JF308542
L. osculatiana De Not.	Panama	Schäfer-Verwimp & Verwimp 30958 (GOET)	KF556538	KF556314	KF556631
L. pallescens Mitt.	Ecuador	Schäfer-Verwimp 32731 (GOET)	KF556540	-----------	KF556069
L. parva (S.Hatt.) Mizut.	Japan	Mizutani 15293 (L)	KF556542	KF556318	KF556072
L. papilionacea Prantl.	São Tomé	Shevock 42335 (EGR)	MN822981	MN823061	MN818961 (ITS2)
L. patersonii (Steph.) Steph.	Thailand	G.E.Lee 2525 (UKMB)	MN822982	MN823042	MN819003
L. patriciae Schäf.-Verw.	Peninsular Malaysia	Pócs et al. 13161/2 (EGR)	MN822983	MN823043	MN819004
L. paucidentata (Steph.) Grolle	Cuba	Pócs & Caluff 9199/CL (JE)	-----------	KF556321	KF556075
L. pectinella Mizut.	Sabah, Borneo	Lee 1672 (UKMB)	MN822984	MN823044	MN819005
L. phyllobola Nees & Mont.	Ecuador	Noeske et al. 204 (GOET)	KF556600	KF556322	KF556076
L. pterigonia (Lehm. & Lindenb.) Mont.	Bolivia	Churchill et al. 21851 (GOET)	KF556546	KF556325	KF556078

L. ptosimophylla C.Massal.	Argentina	Vinocur MER502 (GOET)	-----------	MN823045	MN818964 (ITS1)
L. puiggariana Steph.	Dominican Rep.	Schäfer-Verwimp & Verwimp 27016/A (GOET)	KF556550	KF556329	KF556082
L. pulchriflora (Pearson) G.E.Lee et al.	Tanzania	Pócs 89126/M (EGR)	KT897948	KT897953	KT897943
L. pulverulenta (Gottsche ex Steph.) M.E.Reiner	Bolivia	Reiner-Drehwald & Drehwald 4517 (GOET)	KF556552	KF556331	KF556084
L. ramosissima Steph.	São Tomé and Príncipe	Shevock 34348A (EGR)	KF556554	KF556333	KF556086
L. ramulosa (Herzog) R.M.Schust.	Costa Rica	Schäfer-Verwimp & Holz SV/H-<br>0229/A (GOET)	KF556555	KF556335	KF556088
L. reflexistipula (Lehm. & Lindenb.) Gottsche<br>et al.	Ecuador (III)	Schäfer-Verwimp & Nebel 33162<br>(GOET)	KF556559	KF556340	KF556092
L. reinerae Ilk.-Borg.	Fiji Isl.	Pócs & Pócs 03261/AC (EGR)	MN822985	MN823046	MN819006
L. rotundifolia Mitt.	Costa Rica, Cartago	Schäfer-Verwimp & Holz SV/H- 0378 (GOET)	KF556567	KF556348	KF556099
L. ruthii (A.Evans) R.M.Schust.	USA, Tennessee	Zartman 681 (DUKE)	KF556569	-----------	KF556634
L. sikorae (Steph.) Steph.	Madagascar	Pócs & Szabó 9878/EZ (EGR)	MN822986	MN823047	MN819007
L. soae R.L.Zhu et al.	China	Long 24473b (E)	MN822987	MN823048	MN819008
L. sordida (Nees) Nees	Papua New Guinea	Kumei 15 (JE)	MN822988	MN823049	MN819009
L. sporadica Besch. & Spruce	Panama	Schäfer-Verwimp & Verwimp<br>31033 (GOET)	KF556583	-----------	KF556117
L. stenodentata M.A.M.Renner & Pócs	Papua New Guinea	Streimann 41784 (JE)	MN822989	MN823050	MN819010
L. stephaniana Mizut.	Sabah, Borneo	G.E.Lee 1515 (UKMB)	MN822990	-----------	MN819011
L. stevensiana (Steph.) Mizut.	China	Long 37118 (E)	MN822991	MN823051	MN819012
L. subolivacea Mizut.	Bangladesh	Long 28096 (E)	MN822992	MN823052	MN819013
L. subplana (Steph.) C.Bastos	Brazil	Schäfer-Verwimp & Verwimp 6953/B (M)	MN822993	MN823053	MN819014
L. subspathulata Spruce	Dominican Rep.	Gradstein 6643 (GOET)	KF556585	KF556367	KF556119
L. sulphurea (Lehm. & Lindenb.) Spruce	Guadeloupe	Schäfer-Verwimp 22453 (GOET)	MN822994	MN823054	MN819015
L. tapajosensis Spruce	Ecuador	Nöske et al. 204 (GOET)	KF556589	KF556371	KF556122
L. tasmanica Gottsche et al.	New Zealand	Renner 872054 (NSW)	-----------	JF308581	JF308552
L. terricola Spruce	Argentina	Reiner MER1148 (M)	MN822995	MN823055	MN819016
L. thallophora (Eifrig) Gradst.	Indonesia	Abrahamczyk 05285 (JE)	MN822996	MN823056	MN819017
L. topoensis Gradst. & M.E.Reiner	Ecuador	Gradstein & Jost 10063 (GOET)	DQ983712	DQ987416	DQ987312
L. trinitensis Lindenb. & Gottsche	Brazil	Vital 10.168 (JE)	KF556594	KF556376	KF556127
L. tuberculosa Steph.	São Tomé and Príncipe	Shevock 42453 (EGR)	MN822997	MN823057	MN819018
L. tumida Mitt.	New Zealand	Renner 299949 (AK)	-----------	JF308556	JF308527
L. umbilicata (Nees) Nees et al.	Indonesia	Schäfer-Verwimp & Verwimp 16954 (GOET)	KF556597	KF556379	KF556130

L. urbanii Steph.	Dominican Rep.	Schäfer-Verwimp & Verwimp 27006 (GOET)	MN822998	MN823058	MN819019
L. utriculata (Steph.) Mizut.	Peninsular Malaysia	Schäfer-Verwimp & Verwimp 18900/C (M)	MN822999	MN823059	MN819020
L. wightii Lindenb.	Thailand	Schäfer-Verwimp & Verwimp 23844 (M)	MN823000	MN823004	MN819021
Microlejeunea filicuspis (Steph.) Heinrichs et al.	Fiji Isls.	Pócs & Pócs 03304/A (EGR)	KC313138	KC313178	KC313100
M. latitans (Hook.f & Taylor) Heinrichs et al.	New Zealand	Schäfer-Verwimp & Verwimp 13869 (JE)	KC313146	KC313186	KC313108
